# Concurrent Treatment of Posttraumatic Stress Disorder and Alcohol Use Disorder in Women: A Randomized Clinical Trial

**DOI:** 10.1001/jamanetworkopen.2025.21087

**Published:** 2025-07-15

**Authors:** Anna Persson, Åsa Axén, Andrea Johansson Capusan, Åsa Magnusson, Markus Heilig

**Affiliations:** 1 Centre for Psychiatry Research, Department of Clinical Neuroscience, Karolinska Institutet and The Stockholm Centre for Dependency Disorders, Stockholm, Sweden; 2 Center for Social and Affective Neuroscience, Department of Biomedical and Clinical Sciences, Linköping University, Linköping, Sweden

## Abstract

**Importance:**

Trauma-focused treatments are effective for posttraumatic stress disorder (PTSD) but are rarely offered to patients with comorbid substance use disorder. Research suggests gender-based differences in prevalence and treatment needs for these patients, but treatment trials have mainly included men.

**Objective:**

To evaluate whether integrated trauma-focused psychological treatment (ie, integrated treatment) leads to greater reduction in PTSD symptom severity and weekly alcohol use than usual treatment (ie, relapse prevention) for alcohol use disorder (AUD) in women.

**Design, Setting, and Participants:**

This randomized clinical trial was conducted at 3 outpatient addiction services in Sweden. Data were collected from 2016 to 2021, and participants were followed up for 9 months after treatment initiation. Data were analyzed from October 2024 to April 2025. Participants were women older than 18 years with current PTSD and moderate-to-severe AUD diagnoses meeting *Diagnostic and Statistical Manual of Mental Disorders*, Fifth Edition (*DSM-5*) criteria. Participants were randomly assigned to either the integrated treatment or relapse prevention arm. Intention-to-treat analyses were carried out using linear mixed models.

**Interventions:**

Twelve sessions, typically weekly, of integrated treatment (ie, Concurrent Treatment of PTSD and Substance Use Disorders Using Prolonged Exposure [COPE]) or relapse prevention were delivered by trained and experienced staff (including registered nurses, licensed psychologists, and social workers).

**Main Outcomes and Measures:**

Prespecified co–primary outcomes were PTSD symptom severity (assessed by blinded raters using Clinician-Administered PTSD Scale for *DSM-5* [CAPS-5]) and weekly alcohol use (self-assessed using Timeline Followback) from baseline to the 9-month follow-up. Secondary outcomes included self-reported PTSD symptom severity, clinician-rated PTSD remission, and an objective biomarker of alcohol use (phosphatidylethanol level).

**Results:**

Ninety women (mean [SD] age, 44.7 [12.5] years) were included and randomly assigned to integrated treatment (n = 45) or relapse prevention (n = 45). In both arms, PTSD symptom severity decreased from baseline to 9-month follow-up (mean CAPS-5 score for integrated treatment: 37.40 [95% CI, 33.84-40.96] to 13.18 [95% CI, 8.95-17.41]; relapse prevention: 39.09 [95% CI, 35.53-42.65] to 23.68 [95% CI, 19.47-27.88]), with a significantly greater decrease in the integrated treatment arm than the relapse prevention arm (treatment-by-time interaction: *F*_4,155_ = 3.0; *P* = .02). Self-reported alcohol use decreased significantly over time (*F*_14,581_ = 3.0; *P* < .001) in both arms (integrated treatment: 144.41 [95% CI, 104.66-184.15] g/week to 92.65 [95% CI, 48.81-136.48] g/week; relapse prevention: 133.45 [95% CI, 93.71-173.19] g/week to 77.80 [95% CI, 31.65-123.95] g/week), but there was no detectable difference between treatments.

**Conclusions and Relevance:**

In this trial of integrated treatment vs relapse prevention, integrated treatment led to a greater reduction in PTSD symptom severity and no detectable difference in alcohol use decrease compared with relapse prevention. These results support that integrated treatment can safely and effectively treat PTSD in women with AUD and ongoing alcohol use.

**Trial Registration:**

ISRCTN.org Identifier: ISRCTN61391164

## Key Points

**Question:**

Will integrated trauma-focused psychological treatment lead to greater reductions in posttraumatic stress disorder (PTSD) symptom severity and alcohol use than usual treatment for alcohol use disorder (AUD) in women?

**Findings:**

In this randomized clinical trial of 90 women receiving outpatient addiction services, integrated treatment significantly reduced PTSD symptom severity more than relapse prevention (usual treatment for AUD). Self-reported alcohol use also decreased, with no detectable differences between the treatment arms.

**Meaning:**

The findings suggest that PTSD can be safely and effectively treated using integrated treatment in women with AUD and ongoing alcohol use.

## Teaser

This randomized clinical trial compares the effect of trauma-focused psychological treatment vs relapse prevention on posttraumatic stress disorder symptom severity and alcohol use in women.

## Introduction

Posttraumatic stress disorder (PTSD) has an estimated lifetime prevalence of 3.9%.^1,2^ It is associated with multiple adverse health outcomes^3^ and high societal costs.^3,4,5,6^ PTSD is also associated with subsequent onset of alcohol use disorder (AUD), and vice versa.^7^ People with comorbid PTSD and AUD have higher symptom severity, report more suicide attempts, and use more mental health care than people with either disorder alone.^8^ Individual trauma-focused psychological treatment for PTSD and AUD can reduce PTSD symptom severity and alcohol use for patients with these comorbid disorders, but the evidence is not yet robust and more research is needed.^9,10,11,12,13,14^ In the US, only 18.4% of people with lifetime diagnoses of PTSD and AUD have received treatment for their mental health and AUD.^15^ In Europe, clinicians underdiagnose PTSD among individuals with comorbid PTSD and substance use disorder (SUD) and rarely offer them PTSD treatment.^16,17,18,19,20,21^

Concurrent Treatment of PTSD and Substance Use Disorders Using Prolonged Exposure or COPE (hereafter, integrated treatment) was developed to treat comorbid PTSD and SUD.^22,23^ Integrated treatment combines evidence-based interventions for these conditions into a trauma-focused, manual-based treatment addressing both disorders and their interplay.^23^ Prolonged exposure is used to treat PTSD along with relapse prevention for SUD.^23^ In randomized clinical trials (RCTs), integrated treatment has reduced PTSD symptom severity more than the usual treatment for SUD alone and has reduced substance use as much as SUD treatment in civilian^24,25^ and military veteran populations.^26^ Integrated treatment has also reduced PTSD symptoms more than present-centered integrated coping skills therapy and decreased the percentage of heavy drinking days as much as integrated coping skills therapy among military veterans.^27^ Military populations tend to have worse treatment outcomes than civilians.^28^ In civilians, RCTs on integrated treatment have primarily included participants with SUD and substances other than alcohol.^24,25^ People with PTSD and AUD or SUD may have different treatment needs, as their courses and clinical outcomes differ.^15^

Lifetime PTSD prevalence in women is more than twice that in men (OR 2.6),^1^ and women make up 67.7% of people with comorbid PTSD and AUD.^15^ These findings may be due to higher risks in women of experiencing potentially traumatic events (PTEs) at a younger age and/or experiencing physical and sexual assault, all of which are associated with a higher risk of developing PTSD.^14^ People rarely, spontaneously report having experienced sexual assault, and health care practitioners ask about it less often than other PTEs.^14,29,30,31^ Taken together, these situations may add to PTSD being underdiagnosed and undertreated in women. Despite making up the majority of the patient population, women are underrepresented in treatment research on comorbid PTSD and AUD, with US trials including only 22.0% female participants.^32^ Similarly, 3 of 4 RCTs on integrated treatment predominantly included men.^25,26,27^

Research also indicates gender-based differences in treatment needs and barriers. Women and men may respond differently to psychological treatment of co-occurring disorders.^33^ Among people recovering from SUD, women have more severe PTSD symptoms than men,^34^ women with primary AUD may be at greater risk of relapse to alcohol use due to residual symptoms after AUD treatment,^35^ and gender-specific behavioral treatments may be needed.^34^ There are gender-specific barriers to treatment of comorbid PTSD and AUD, with women more than men reporting that their mental health (eg, PTSD symptoms) poses a barrier.^34^

We therefore set out to evaluate the efficacy of integrated treatment in women with current PTSD and moderate-to-severe AUD diagnoses meeting the *Diagnostic and Statistical Manual of Mental Disorders*, *Fifth Edition* (*DSM-5*) criteria.^36^ To our knowledge, this trial is the first to investigate the efficacy of integrated treatment outside of the English language sphere. Based on prior research,^24,25,26,37^ our prespecified co–primary objective was to evaluate whether integrated treatment leads to greater reduction in PTSD symptom severity and weekly alcohol use than usual treatment (ie, relapse prevention) for AUD. Our secondary objective was to investigate whether integrated treatment is superior to relapse prevention regarding self-reported PTSD symptoms; clinician-rated PTSD remission; and phosphatidylethanol level, an objective biomarker of alcohol use.

## Methods

### Design

We conducted a parallel group RCT with 1:1 allocation to integrated treatment vs relapse prevention in women with current *DSM-5*–defined PTSD and AUD diagnoses. Data were collected from July 13, 2016, to February 25, 2021. The Regional Ethical Review Board in Stockholm approved the trial. Trial protocol and statistical analysis plan are provided in Supplement 1. Participants provided written informed consent. We followed the Consolidated Standards of Reporting Trials (CONSORT) reporting guideline.

### Setting

The study was carried out at 3 outpatient addiction services in Sweden—2 in Stockholm (population of approximately 1.7 million) and 1 in Linköping (population of approximately 170 000). Two of these sites were funded and operated by the regional government, similar to most Swedish health care. The third site was publicly funded but operated by a private health care organization.

### Participants

Women seeking outpatient treatment for PTSD or AUD at the study sites were assessed for eligibility. A psychologist or psychiatrist (including A.J.C., Å.M., and A.P.) administered the Life Events Checklist for *DSM-5* to screen for PTEs.^38^ Eligibility was also evaluated using the Mini International Neuropsychiatric Interview for *DSM-5* and clinical examination.^39^

Inclusion criteria were woman gender, age older than 18 years, and current *DSM-5*–defined PTSD and moderate-to-severe AUD diagnoses. Exclusion criteria were current moderate-to-severe SUD other than nicotine; clinically significant suicidal or homicidal ideation; ongoing medication that could influence study outcomes (eg, AUD medication); insufficient memory of the trauma for prolonged exposure to be effective; dissociative disorder that was more severe or had greater effect than her PTSD; or any other condition that, in the judgment of the investigator, made participation in the study as not being in the best interests of the individual. We excluded AUD medication due to its lower effectiveness and tolerability and worse adverse effects among women than men,^40^ the population’s varying goals (including abstinence and reduced alcohol use), and to enable us to run a 2-arm RCT with adequate power.

Eligible participants were offered verbal and written information about the trial by the health care staff. Those who were interested in participating met with a psychiatrist for additional verbal and written information about the trial before providing written informed consent. Once consent was obtained, any ongoing medication was kept constant for 8 weeks, except for AUD medications, which had to be discontinued at least 2 weeks before baseline assessment. Assessment instruments included the Clinician-Administered PTSD Scale for *DSM-5* (CAPS-5; score range: 0-80, with higher scores indicating greater severity),^41^ PTSD Checklist–Civilian Version (PCL-C; score range: 17-85, with higher scores indicating greater severity),^42^ Timeline Followback (TLFB),^43^ and the alcohol use biomarker phosphatidylethanol.^44^ For a complete list of assessments used, see eTable 1 in Supplement 2 or Supplement 1.

All participants received a 50 Sk (Swedish krona; approximately US $5) gift card after sessions 1 to 12 and a 150 Sk (approximately $16) gift card after follow-up visits. Gift cards could be redeemed at a department store.

### Interventions

Integrated treatment is designed to be delivered individually over 8 to 15 sessions lasting 60 to 90 minutes each.^23^ It combines prolonged exposure with relapse prevention, encompassing psychoeducation on PTSD, SUD, and their interplay; imaginal and in vivo exposure to treat PTSD; and skills to address SUD (eg, identifying and addressing craving; anger awareness and management).^23^ Participants were scheduled to receive 12 individual sessions lasting 60 to 90 minutes each, typically weekly, based on a pilot study in the same population.^45^ Existing staff (registered nurses, licensed psychologists, and social workers) completed 3 days of training prior to delivering integrated treatment. Staff received supervision every 2 weeks by psychologists with extensive training in prolonged exposure and who were certified by the Center for the Treatment and Study of Anxiety at the University of Pennsylvania. Staff had 0 to 4 years of experience providing integrated treatment before the trial. Information about integrated treatment is available in the therapist guide.^23^

Relapse prevention focuses on engendering skills that are useful for SUD recovery, such as becoming aware of craving and dealing with it in healthy ways, managing thoughts about substance use, and developing refusal skills. We used the Project MATCH Cognitive-Behavioral Coping Skills Therapy Manual.^46^ Participants were scheduled to receive 12 individual sessions lasting 45 to 60 minutes each, typically weekly, specifically including the 8 core sessions and the elective sessions on assertiveness, managing negative moods and depression, and anger awareness and management. Existing staff (registered nurses and social workers), with several years of experience of providing relapse prevention, completed 1 day of additional training before delivering relapse prevention. Information about relapse prevention can be found in the manual.^46^

Participants set their own alcohol-related goals, including abstinence and reduced use. They could also change these goals during treatment.

All sessions were video recorded. For integrated treatment, 7.5% of sessions were monitored for fidelity by a prolonged exposure trainer. Fidelity ratings based on a scale (range: 0-4) used in previous studies^26,27^ indicated very good treatment adherence (mean [SD] rating, 3 [0.4]). For relapse prevention, 24.4% of sessions were monitored for fidelity by a licensed psychotherapist trained in relapse prevention. Fidelity ratings based on a previously developed scale^47^ (range: 0-5) indicated adequate to good treatment adherence (mean [SD] rating, 3.6 [0.9]). There was no contamination across intervention groups, and no treatment modifications. Treatment was to be delivered over a maximum of 20 weeks.

The 3 study sites randomly assigned 67, 7, and 16 participants to the treatment arms. All sites provided integrated treatment and relapse prevention. Staff with qualifications required by the treatment manuals^23,46^ were trained in the interventions. At 1 site, the availability of staff qualified to deliver integrated treatment was low; hence, qualified staff delivered integrated treatment only. Six staff members delivered integrated treatment only, 5 delivered relapse prevention only, and 3 delivered both integrated treatment and relapse prevention. Staff treated 1 to 20 participants.

Integrated treatment and relapse prevention are individually delivered cognitive behavior therapies. Both include relapse prevention and, in this study, sessions on anger awareness and management, which are not always included in relapse prevention. Integrated treatment addresses both PTSD and AUD or SUD,^23^ and relapse prevention addresses AUD or SUD.^46^ Both treatments have considerable attrition rates.^9,10,13,48,49^ Completion of psychological treatment of comorbid PTSD and SUD is between 50.4% and 70.6%.^9,48^ Mean dropout rates were 26.1% from in-person psychosocial AUD treatment, 30.2% from treatment with a greater number of sessions (14 sessions, similar to the 12 sessions of integrated treatment and relapse prevention), and 37.0% from studies using a *DSM* diagnosis to confirm SUD (such as this study).^49^ No adjunctive treatments were offered as part of this trial, but participants were free to access any other treatment.

### Randomization and Blinding

A third party, Karolinska Trial Alliance, handled randomization using a computer-generated sequence with permuted blocks of 6. No stratification was used. The allocation sequence was concealed in sealed envelopes. After baseline assessment, staff opened the envelopes in numerical order to assign participants to integrated treatment or relapse prevention. Neither researchers nor trial staff had access to the randomization sequence or block size during the trial. Baseline assessment was completed before participants were allocated to treatment. Thus, staff and participants were unaware of allocation at that point in time. After randomization, participants and trial staff delivering the treatment were aware of allocation, but raters assessing PTSD severity were blinded.

### Outcomes

A co–primary outcome was PTSD symptom severity, assessed with CAPS-5,^41^ after sessions 6 and 12 and at the 6- and 9-month follow-up. The CAPS-5 is the gold standard measure of PTSD^50^ and was conducted by trained interviewers blinded to treatment allocation. The other primary outcome was self-reported alcohol use, which was measured as grams per week using TLFB^43^ at each session and at the 6- and 9-month follow-up. For reference, US and Swedish standard drinks contain 14 and 12 g of alcohol, respectively.^51^

Secondary outcomes were self-rated PTSD symptom severity; clinician-rated PTSD remission; and phosphatidylethanol level, a biomarker of alcohol use. Because no adult *DSM-5*–defined PTSD self-report measure was available in Swedish when the trial started, we used PCL-C^42^ at each session and the 6- and 9-month follow-up. Clinician-rated PTSD remission also used CAPS-5. Phosphatidylethanol^44^ level in blood was measured at sessions 6 and 12 and the 6- and 9-month follow-up. Follow-ups were changed from 6 and 9 months after baseline to 6 and 9 months after treatment start before the analysis commenced.

### Power Analysis and Sample Size

To allow detection of a clinically meaningful medium effect size, Cohen *d* = 0.5, with 80% power at α = .05, we intended to include 150 participants for approximately 120 completers. The trial was terminated at 90 participants because accrual rates indicated that it would not be possible to complete it within a feasible time frame. No interim analyses were planned or conducted.

### Statistical Analysis

Data were analyzed from October 2024 to April 2025. Preselected linear mixed models that included treatment, time, and treatment-by-time interaction as fixed effects and participants as a random effect and a random intercept were run in SPSS, version 28 (IBM).^52^ Restricted maximum likelihood estimation, Satterthwaite approximation, and a first-order autoregressive covariance matrix were used.

Intention-to-treat (ITT) analyses using data from all randomly assigned participants are presented along with modified ITT analyses excluding 2 participants who had PTSD symptoms but not current *DSM-5*–defined PTSD at baseline. The amount of missing data was 17.2%. Little χ^2^ statistic indicated that the data were missing completely at random (*P* > .05). Study staff tried to reach participants twice to reschedule when they did not show up for sessions or follow-up visits. All participants were asked to complete follow-up visits. Two-sided *P* < .05 indicated statistical significance.

## Results

**Figure 1.**
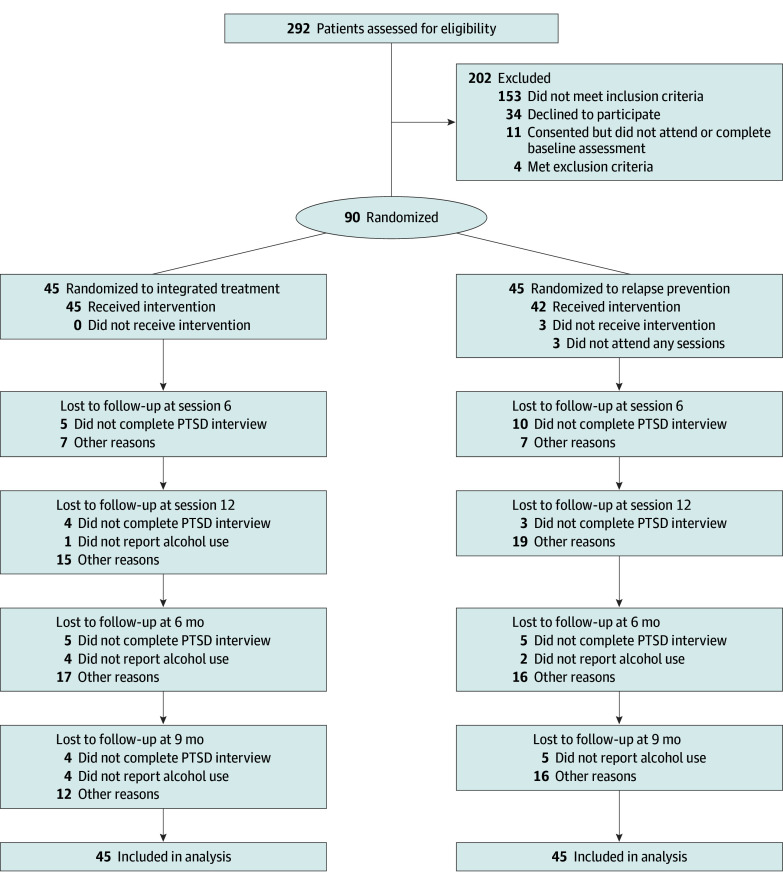
CONSORT Flow Diagram PTSD indicates posttraumatic stress disorder. For additional details, see the eFigure in Supplement 2.

All 292 women seeking treatment at the 3 addiction services were assessed for eligibility, of whom 157 were excluded, 34 declined to participate, and 11 consented but did not participate (Figure 1). Ninety women (mean [SD] age, 44.7 [12.5] years) were randomly assigned to integrated treatment (n = 45) or relapse prevention (n = 45) and included in the analysis (Table 1). Twenty-one women (23.3%) were born outside Sweden (the national figure was 20.9%).^53^ For participant disposition, see Figure 1; greater details are provided in the eFigure in Supplement 2. For baseline characteristics of participants, see Table 1.

**Table 1.** Baseline Characteristics of Participants

Characteristics	Participants, No. (%)		
	Total (n = 90)	Integrated treatment (n = 45)	Relapse prevention (n = 45)
Demographic			
Women gender	90 (100)	45 (100)	45 (100)
Age, mean (SD), y	44.7 (12.5)	45.1 (13.0)	44.4 (12.2)
Born in Sweden	68 (75.6)	31 (68.9)	37 (82.2)
Born in another country	21 (23.3)^a^	13 (28.9)	8 (17.8)
Educational level, highest completed			
Upper secondary school	30 (33.3)	14 (31.1)	16 (35.6)
Some university	39 (43.3)	21 (46.7)	18 (40.0)
Occupational status			
Employed	42 (46.7)	19 (42.2)	23 (51.1)
Sickness benefit	33 (35.6)	16 (35.6)	17 (37.8)
Relationship status			
Single	63 (70.0)	33 (73.3)	30 (66.7)
Married or cohabiting	25 (27.8)	10 (22.2)	15 (33.3)
Trauma history			
Age at first trauma, median (minimum-maximum), y	11 (2-49)	12 (2-49)	10 (3-42)
Experienced trauma during childhood	58 (64.4)	29 (64.4)	29 (64.4)
Experienced sexual abuse during childhood	37 (41.1)	16 (35.6)	21 (46.7)
Time since index trauma, median (minimum-maximum), y	13 (0-70)	10 (1-60)	16 (0-70)
No. of trauma types experienced, mean (SD)	6.9 (2.6)	6.4 (2.4)	7.4 (2.6)
Type of index trauma			
Physical assault	28 (31.1)	12 (26.7)	16 (35.6)
Sexual assault	27 (30.0)	9 (20.0)	18 (40.0)
Assault with a weapon	8 (8.9)	6 (13.3)	2 (4.4)
Sudden accidental death	7 (7.8)	4 (8.9)	3 (6.7)
Other	17 (18.9)	13 (28.9)	4 (8.9)
PTSD			
Duration of PTSD symptoms, median (minimum-maximum), y	8 (0-68)	6 (0-59)	10 (0-68)
Delayed onset	29 (32.2)	13 (28.9)	16 (35.6)
Dissociative symptoms	16 (17.8)	6 (13.3)	10 (22.2)
Prior PTSD treatment	13 (14.4)	5 (11.1)	8 (17.8)
AUD			
Duration of AUD symptoms, median (minimum-maximum), y	6 (0-47)	7 (0-47)	6 (1-42)
Prior AUD treatment	47 (52.5)	24 (53.3)	23 (51.1)
Other mental health history			
Current psychiatric diagnoses, in addition to PTSD and AUD	56 (62.2)	28 (62.2)	28 (62.2)
Currently taking psychotropic medication	71 (78.9)	34 (75.6)	37 (82.2)
Attempted suicide			
Lifetime	32 (35.6)	13 (28.9)	19 (42.2)
Past year	9 (10.0)	4 (8.9)	5 (11.1)
Baseline assessment scores			
CAPS-5 score, mean (SD)^b^	38.2 (10.4)	37.4 (10.8)	39.1 (10.1)
Alcohol use			
Grams of alcohol consumed per wk in past 90 d, median (minimum-maximum)^c^	93.9 (0-531.0)	108.0 (0-480.0)	84.9 (0-531.0)
Percent of heavy drinking days in past 90 d, median (minimum-maximum)	12.5 (0-80.6)	18.4 (0-80.6)	9.4 (0-77.1)

Abbreviations: AUD, alcohol use disorder; CAPS-5, Clinician-Administered PTSD Scale for *Diagnostic and Statistical Manual of Mental Disorders*, Fifth Edition; PTSD, posttraumatic stress disorder.

^a^ Nationally, 20.9% of the women were born in another country.^53^

^b^ CAPS-5 score range: 0 to 80, with higher scores indicating greater severity.

^c^ For the total sample, integrated treatment arm, and relapse prevention arm, this amount corresponds to approximately 6.7, 7.7, and 6.1 US standard drinks containing 14 g of alcohol and to 7.8, 9.0, and 7.1 Swedish standard drinks containing 12 g of alcohol.^51^

Participants in the integrated treatment and the relapse prevention arms attended a median (minimum-maximum) of 12 (1-12) and 12 (0-12) sessions, respectively. Mean (SD) treatment length was 14.8 (6.6) weeks for the integrated treatment group and 14.2 (5.6) for the relapse prevention group. Fifty-six participants (62.2%) completed 12 sessions, 12 (13.3%) completed fewer sessions but did not drop out, and 22 (24.4%) dropped out. Kaplan-Meier analysis and a log rank test indicated that there was no detectable difference in dropout distributions between treatment groups.

Overall, 58 participants (64.4%) experienced trauma during childhood, and a mean (SD) of 6.9 (2.6) trauma types were experienced. The median (minimum-maximum) duration of symptoms was 8 (0-68) years for PTSD and 6 (0-47) years for AUD. Fifty-six participants (62.2%) had current psychiatric diagnoses in addition to PTSD and AUD (Table 1).

### PTSD Outcomes

**Figure 2.**
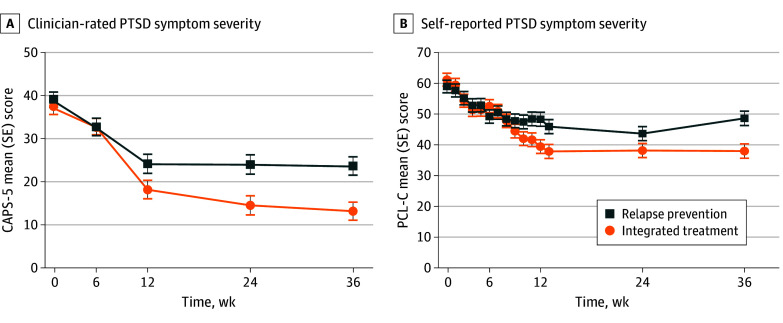
Clinician- and Self-Rated Posttraumatic Stress Disorder (PTSD) Symptom Severity A, Symptom severity assessed by blinded raters using Clinician-Administered PTSD Scale for *Diagnostic and Statistical Manual of Mental Disorders*, Fifth Edition (CAPS-5; score range, 0-80, with higher scores indicating greater severity) supported superiority of integrated treatment (treatment-by-time interaction: *P* = .02). B, Superiority of integrated treatment was supported by self-reported symptom severity using PTSD Checklist–Civilian Version (PCL-C; score range: 17-85, with higher scores indicating greater severity) (treatment-by-time interaction: *P* < .001). Error bars represent SEs.

Clinician-rated PTSD symptom severity decreased in both arms from baseline to 9-month follow-up (mean CAPS-5 score, integrated treatment: 37.40 [95% CI, 33.84-40.96] to 13.18 [95% CI, 8.95-17.41]; relapse prevention: 39.09 [95% CI, 35.53-42.65] to 23.68 [95% CI, 19.47-27.88]), with a significantly greater decrease in the integrated treatment arm than the relapse prevention arm (treatment-by-time interaction: *F*_4,155_ = 3.0; *P* = .02) (Figure 2A, Table 2).

**Table 2.** Continuous Outcomes at Baseline, After Treatment, and Follow-Up

Outcome and time point	Estimated marginal mean from linear mixed models (95% CI)	
	Integrated treatment	Relapse prevention
PTSD symptom severity		
CAPS-5 score^a^		
Baseline	37.40 (33.84-40.96)	39.09 (35.53-42.65)
After treatment	18.25 (14.01-22.49)	24.25 (19.85-28.65)
6-mo Follow-up	14.55 (10.12-18.99)	24.09 (19.73-28.45)
9-mo Follow-up	13.18 (8.95-17.41)	23.68 (19.47-27.88)
PCL-C score^b^		
Baseline	61.27 (57.15-65.40)	59.04 (54.93-63.16)
After treatment	37.99 (33.51-42.47)	46.03 (41.49-50.57)
6-mo Follow-up	38.32 (33.74-42.91)	43.79 (39.25-48.34)
9-mo Follow-up	38.12 (33.58-42.66)	48.75 (44.09-53.41)
Alcohol use		
Grams per wk		
Baseline	144.41 (104.66-184.15)	133.45 (93.71-173.19)
After treatment	96.84 (52.99-140.68)	72.27 (27.48-117.06)
6-mo Follow-up	88.28 (42.42-134.14)	99.41 (54.69-144.14)
9-mo Follow-up	92.65 (48.81-136.48)	77.80 (31.65-123.95)
PEth level		
Baseline	0.68 (0.49-0.88)	0.45 (0.26-0.65)
After treatment	0.61 (0.39-0.82)	0.34 (0.11-0.56)
6-mo Follow-up	0.57 (0.36-0.78)	0.44 (0.23-0.65)
9-mo Follow-up	0.69 (0.44-0.93)	0.29 (0.04-0.54)

Abbreviations: CAPS-5, Clinician-Administered PTSD Scale for *Diagnostic and Statistical Manual of Mental Disorders*, Fifth Edition; PCL-C, PTSD Checklist–Civilian Version; PEth, phosphatidylethanol; PTSD, posttraumatic stress disorder.

^a^ CAPS-5 score range: 0 to 80, with higher scores indicating greater severity.

^b^ PCL-C score range: 17 to 85, with higher scores indicating greater severity.

Self-reported PTSD symptom severity also decreased in both arms, with a significantly greater decrease in the integrated treatment arm than the relapse prevention arm (treatment-by-time interaction: *F*_14 520_ = 2.7; *P* < .001) (Figure 2B, Table 2). Modified ITT analyses yielded similar results. For clinician-rated PTSD remission results, see eTable 2 in Supplement 2.

### AUD Outcomes

**Figure 3.**
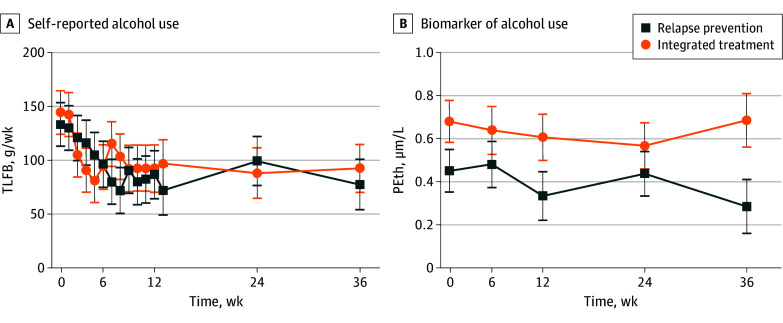
Alcohol Use A, Self-reported weekly alcohol use was assessed with the Timeline Followback (TLFB) instrument. There was a significant overall decrease in use (*P* < .001), but neither the main treatment effect nor the treatment-by-time interaction was significant (all *P* > .05). B, The objective blood biomarker, phosphatidylethanol (PEth), did not support an overall reduction in use or a treatment effect (*P* > .05). Error bars represent SEs.

There was a significant decrease in self-reported alcohol use over time (*F*_14,581_ = 3.0; *P* < .001) in both integrated treatment (144.41 [95% CI, 104.66-184.15] g/week to 92.65 [95% CI, 48.81-136.48] g/week) and relapse prevention (133.45 [95% CI, 93.71-173.19] g/week to 77.80 [95% CI, 31.65-123.95] g/week), but there were no statistically significant effects of treatment or treatment-by-time interaction (Figure 3A, Table 2).

There were no detectable effects of treatment, time, or their interaction on phosphatidylethanol levels (Figure 3B, Table 2). Modified ITT analyses yielded similar results.

### Safety and Tolerability

One participant randomly assigned to the integrated treatment group died in an accident during the trial. There were no treatment emergent serious adverse events during the trial.

## Discussion

In this trial, integrated treatment significantly reduced clinician-rated and self-reported PTSD symptom severity compared with relapse prevention. This finding supports our hypotheses that integrated treatment would be associated with a significantly greater reduction in PTSD symptom severity than relapse prevention. Integrated treatment and relapse prevention were both associated with significant reduction in self-reported weekly alcohol use but not with phosphatidylethanol levels, and there were no detectable differences between treatment groups; hence, our hypothesis that integrated treatment would be associated with significantly greater reductions in alcohol use and phosphatidylethanol levels was not supported.

Integrated treatment was developed to treat comorbid PTSD and SUD and has produced promising results in multiple studies.^22,23,24,25,26,27^ The present trial extends this evidence to women with PTSD and AUD, a group that is overrepresented among people with PTSD and AUD yet underrepresented in treatment trials.^8,32^ It is also, to our knowledge, the first RCT to support the efficacy of integrated treatment in translation (English to Swedish).

Integrated treatment and relapse prevention were both associated with a reduction in PTSD symptom severity, but the reduction was significantly greater for integrated treatment, in line with findings of previous research on comorbid PTSD and SUD treatment.^12^ Research has indicated that improvement in PTSD symptoms has a greater impact on alcohol use than the effect of reduced alcohol use on PTSD,^37^ a notion supported by an earlier RCT investigating integrated treatment.^26^ Hence, we hypothesized that integrated treatment compared with relapse prevention would be associated with a significantly greater decrease in alcohol use, whether self-reported or indicated by phosphatidylethanol level. However, data did not support this hypothesis, consistent with several other integrated treatment trials.^24,25,27^

Participants in our study received gift cards on completing each visit, and treatment attendance was similar to that previously reported.^9,48,49^ In a pilot study, no financial incentives were provided, and 68.2% of the participants completed all 12 COPE sessions,^45^ similar to the 62.2% completion rate in the present trial. This finding suggests that the financial incentives played a minor, if any, role in participant retention in treatment.

Except for the compensation, our study was highly naturalistic. It had broad inclusion criteria, had few exclusion criteria, and relied on existing staff to provide treatment after minimal training. Participants were typical of treatment-seeking women in addiction services. They had often experienced their first PTE in childhood, had experienced several types of PTEs, and lived with PTSD for years before being diagnosed and offered treatment. They also had multiple psychiatric comorbidities in addition to PTSD and AUD. Lasting beneficial effects of integrated treatment on PTSD symptom severity were found even under these conditions, suggesting that these results will generalize outside the context of an RCT.

### Future Research

To serve individuals with comorbid PTSD and AUD, future research should include groups underrepresented in RCTs^32^ and investigate integrated treatment given as intensive outpatient treatment to potentially increase treatment completion among civilians. This approach is similar to that used among military veterans, a population in which this work has already started.^54^

### Limitations

This study has several limitations. Based on power analysis using effect sizes from prior studies, we planned to include 150 participants but had to terminate at 90 participants. We were nevertheless able to detect beneficial effects of integrated treatment on PTSD, but we may have too few observations to detect potential differences in alcohol use between the 2 treatments. Another potential limitation is the extent of interaction between staff and participants. We delivered both treatments according to their manuals, resulting in longer session duration for integrated treatment than for relapse prevention. Hence, we cannot rule out the possibility that some of the differences detected may be driven by session length rather than specific treatment effects. Finally, the trial’s external validity could have been strengthened by allowing for variable treatment lengths, such as 8 to 15 sessions of integrated treatment or relapse prevention.^23,45^

## Conclusions

This trial indicates that integrated treatment for comorbid PTSD and SUD significantly reduces PTSD symptom severity in women with PTSD and AUD and ongoing alcohol use and is associated with similar reductions in alcohol use as relapse prevention. This trial addresses the fact that women are underrepresented in treatment research and provides data on the safety and effectiveness of integrated treatment in this population. Additionally, the trial shows that integrated treatment retains its efficacy when translated from English to Swedish.

## Notes

Supplement 1.
Trial Protocol and Statistical Analysis Plan

Supplement 2. **eTable 1.** Complete List of Assessments **eTable 2.** Clinician-Rated PTSD Remission **eFigure.** CONSORT Flow Diagram
eReferences

Supplement 3.
Data Sharing Statement
