# Error in the Visual Abstract

**DOI:** 10.1001/jamanetworkopen.2025.32737

**Published:** 2025-08-21

In the Original Investigation titled “Concurrent Treatment of Posttraumatic Stress Disorder and Alcohol Use Disorder in Women: A Randomized Clinical Trial”^1^ published online on July 15, 2025, there was an error in the Intervention section of the Visual Abstract. Under the number of patients randomized, the sentence should be as follows: “Participants were randomized to receive 12 individual sessions of treatment.” This article was corrected.^1^
