# Supplementary material for: Concurrent Treatment of Posttraumatic Stress Disorder and Alcohol Use Disorder in Women: A Randomized Clinical Trial
Source: JAMA Netw Open. 2025 Jul 15;8(7):e2521087. doi: 10.1001/jamanetworkopen.2025.21087 (PMC12371515; doi:10.1001/jamanetworkopen.2025.21087)
Supplement: Supplement 1. — Trial Protocol and Statistical Analysis Plan [file jamanetwopen-e2521087-s001.pdf]

A randomized controlled trial of Concurrent treatment of PTSD and Substance Use Disorders using Prolonged Exposure (COPE) and Relapse Prevention (RP) in women with PTSD and Alcohol Use Disorder.

Sponsor / investigator: Dr. Åsa Magnusson, MD, PhD
Department of Clinical Neuroscience, Karolinska Institutet
EWA-unit, The Stockholm Centre for Dependency Disorders
Tideliusgatan 26, Katarinahuset, 1 tr
118 69 Stockholm
SWEDEN
+46 8 12345 819, +46 12345 840
asa.magnusson@ki.se

Location of trial: EWA-unit, The Stockholm Centre for Dependency Disorders, Tideliusgatan 26, 118 69 Stockholm, Sweden

Capio Maria, Wollmar Yxkullsgatan 25, 118 91 Stockholm, Sweden

The Substance Use Disorders and Psychiatric Clinics, Linköping University Hospital, 581 85 Linköping, Sweden

Summary

Background: Posttraumatic stress disorder (PTSD), a disorder triggered by severe trauma, is characterized by intrusive recollections, avoidance, hyper-arousal and negative changes in cognition and mood. Lifetime PTSD prevalence in women is twice that in men (8-16% vs 4-8). PTSD is highly co-morbid with alcohol dependence (AD), also referred to as moderate to severe alcohol use disorder (AUD). Animal data suggest shared neural substrates for PTSD and AD, and point to an involvement of brain circuitry subserving fear learning and extinction. Prolonged exposure aimed at extinction of trauma related anxiety is an effective behavioral treatment for PTSD, and was recently adapted for use in substance dependent populations (“Concurrent Treatment of PTSD and Substance Use Disorders Using Prolonged Exposure" (COPE)). Different types of traumatic events carry different risks of developing PTSD. Interpersonal violence is associated with the highest PTSD risk and sexual assault is the most common stressor preceding PTSD development in women. PTSD is highly over-represented among patients seeking treatment for AUD, and this co-morbidity represents a large unmet medical need. PTSD is more severe in individuals with substance use disorders, while the risk for relapse is higher among patients in whom PTSD and SUD are co-morbid. There is a need to improve treatment for PTSD and co-morbid SUD beyond currently available options.

Objectives: The overall aim of this research project is to evaluate and, if suitable, implement a novel method for concurrent treatment of co-morbid posttraumatic stress disorder (PTSD) and substance use disorders (SUD) in out-patient care for women with PTSD and moderate to severe alcohol use disorder (AUD), previously referred to as alcohol dependence.
The method we evaluate in this study is a manual based treatment for co-morbid PTSD and SUD: “Concurrent Treatment of PTSD and Substance Use Disorders Using Prolonged Exposure" (COPE), a type of trauma focused cognitive behavioral therapy (CBT) with exposure, integrated with behavioral techniques for relapse prevention (RP). RP is another form of manual based treatment for SUD that is frequently used in the treatment of AUD. The planned study evaluates the hypothesis that COPE will lead to greater reductions in PTSD and AUD symptom severity than RP.

Study population: Up to one hundred and fifty treatment-seeking women, aged >18, diagnosed with DSM-5 PTSD and moderate to severe AUD.

Method: The study consists of three consecutive phases for each patient: *A screening and randomization phase*, carried out over the course of three to four visits, including diagnostic assessment and randomization 1:1 to receive COPE or RP, *a phase with cognitive and*

behavioral therapy, 12 sessions of COPE or 12 sessions of RP, typically one a week, and *a posttreatment follow up*, at session 12, and six and nine months post baseline.

Outcomes: The co-primary outcomes will be 1) change from baseline in PTSD symptom severity (Clinician-Administered PTSD Scale (CAPS-5)), measured after sessions 6 and 12 and six and nine months post baseline. 2) change from baseline in alcohol consumption per week (grams per week) and heavy drinking days (HDD) derived from Timeline Follow Back (TLFB), after therapy sessions 6 and 12 and six and nine months post baseline.

Background

PTSD is common, and lifetime prevalence in the USA is approximately 6.8%. PTSD is highly over-represented among patients seeking treatment for SUD, and more severe in individuals with SUD and co-morbid PTSD (1-6). Lifetime PTSD prevalence in women is approximately twice that in men (8-16% vs. 4-8% in US samples) (3). Among traumatic events, interpersonal violence is associated with the highest risk of developing PTSD (1). The strong association of PTSD and AUD suggests potential overlaps in underlying pathophysiology.

Specifically, pathologically up-regulated amygdala activity following trauma exposure is a consistent finding in PTSD, and is likely to contribute to negatively reinforced alcohol use ("relief drinking"). Conversely, excessive alcohol consumption is likely to further up-regulate amygdala activity, leading to a worsening of PTSD symptoms (6-8). PTSD symptoms are thought to reflect stress-induced changes in neurobiological systems. In particular, impaired extinction of fear is a consistent finding in PTSD. In contrast, extinction involves new learning, mediated by the ventromedial prefrontal cortex (PFC) and its projections to the amygdala (6, 8-10). A hypothesis of the current proposal is that treatment interventions with an ability to promote extinction of trauma-related fear and anxiety will not only lead to reduced PTSD symptom severity, but also be beneficial in reducing heavy alcohol use. Extinction learning per se has been exploited for therapeutic use in PTSD. An implementation of this approach is a form of trauma-focused cognitive behavioral therapy (CBT), a manual based treatment that uses imaginal as well as in vivo exposure to trauma associated stimuli, with the objective of promoting extinction learning. This therapy, "Prolonged Exposure" (PE), has shown positive results in controlled PTSD trials, and has been evaluated in a population with co-morbid PTSD and AUD (11). PE was recently integrated with established behavioral techniques for relapse prevention into a manual based treatment for co-morbid PTSD and SUD, "Concurrent Treatment of PTSD and Substance Use Disorders Using Prolonged Exposure" (COPE; (12)). A recent RCT evaluated COPE in a population of patients with co-morbid PTSD and SUD. In this study, a beneficial effect of COPE on PTSD symptoms was observed compared to a "treatment as usual" control condition. COPE did not have significant beneficial effects on drug use, but similar to (11), this study provided evidence that exposure based treatments are safe in patients with substance use disorders, alleviating common concerns that distress triggered by exposure to trauma associated stimuli might promote relapse to drug use in this patient population (13). Integrated treatment for comorbid disorders has been found to be superior compared to treatment of individual disorders with separate treatment plans and Socialstyrelsen, in their guidelines from 2015, recommends integrated treatment (14).

However, in standard clinical practice, PTSD and SUD are typically treated sequentially, so that patients first receive treatment for their SUD and are required to achieve sobriety before being offered PTSD treatment, typically through referral to a different care provider. This strategy is not consistent with evidence that substance use disorders and psychiatric co-morbidities including PTSD should be treated in an integrated manner (4, 15).

Study objectives

Primary objectives

The co-primary objectives of the study are to determine whether COPE, in women with co-morbid PTSD and moderate to severe AUD, will:

-Reduce PTSD symptoms, measured as change from baseline, to post session 6, and 12, and six and nine months after baseline, compared to RP (measured using the Clinician-Administered PTSD Scale (CAPS))?

-Reduce alcohol use, measured as change from baseline in alcohol consumption per week (grams per week) and heavy drinking days (HDD) from baseline to session 6, and 12, and six and nine months after baseline, compared to RP?

Secondary and exploratory objectives

- To determine whether COPE, compared to RP, will decrease biomarkers of alcohol use and a biomarker of stress.
- To investigate the association between genetic variation and treatment response.
- To investigate if treatment affects levels of functioning in important areas such as work and relationships at six and nine months post baseline.
- To investigate if treatment affects health care consumption.

Subjects

A maximum of 150 subjects will be recruited for a total of 120 completers. Based on a pilot study, we expect attrition rates to be <25%. Subjects that do not provide complete outcome data will be replaced, such that, based on the expected loss, approximately 150 individuals should be included in the study. Subjects will be treatment seeking women aged ≥18 years, diagnosed with co-morbid PTSD and moderate to severe AUD, and eligible according to the following criteria:

Inclusion criteria

1) Female
2) Age ≥18 years
3) Diagnosed with current PTSD and current moderate to severe AUD according to the *Diagnostic and Statistical Manual of Mental Disorders*-5 *(DSM-5)*, as determined by The Mini International Neuropsychiatric Interview, MINI for DSM-5 (16) and clinical examination.

Exclusion criteria

1) Current DSM-5 moderate to severe SUD other than nicotine, as determined by MINI and clinical examination and negative urine screen for illicit drugs.
2) Clinically significant suicidal or homicidal ideation on clinical assessment with the relevant section of MINI (16) and clinical examination.
3) Current medication which may affect the outcome of the study, primarily for AUD,
4) Insufficient memory of the trauma (for exposure therapy to be effective),
5) Dissociative disorder which is more severe or affects the subject more than her PTSD,
6) Any physical or mental illness or condition that affects the patient so that is deemed by her physician not to be in her best interests to participate in the study,
7) IQ < 70.

Study design and methods

Overview

Assessment in clinical practice
Current PTSD and moderate to severe AUD, according to DSM-5 (MINI, LEC, PCL-C, clinical assessment)

Study start
Information
Informed consent, verbal and written

Inclusion criteria

- Female
- Aged 18 years or older
- Current PTSD and moderate to severe AUD, according to DSM-5 and clinical assessment (see Compton & Grant DSM-IV vs. DSM-5).

Exclusion criteria

- Current moderate to severe SUD, other than alcohol and nicotine, according to DSM-5
- Current or not stably treated psychosis
- Sui- or homicidal ideation deemed to be in need of treatment before study treatment can start
- Current medication, which may affect the study outcome, and which is deemed impossible to discontinue for the duration of the study, primarily AUD medication
- Insufficient memory of the trauma (assessed using the CAPS-5)
- Dissociation which is more difficult or affects the subject more than her PTSD
- Somatic or psychiatric illness where it is deemed to not be in the subject's best interest to participate in the study
- IQ < 70

Medication for psychiatric illness to be kept stable for eight weeks prior to baseline assessment and during the 12 weeks of treatment.
Medication for AUD not to be used two weeks prior to baseline assessment or during the twelve weeks of treatment.

Screening, after 8 weeks of stable levels of any medication (week 0)
Interview and assessments
Demographic data, PTSD and alcohol use (structured interview)
PTSD diagnosis and symptom severity (CAPS-5, PCL-C)
Anxiety symptom severity (BAI or STAI)
Depression symptom severity (BDI-II)
Alcohol consumption during the past 90 days (TLFB)
Alcohol craving (PACS)
Alcohol dependence severity (ADS, AUDIT)
Level of function in important areas of life (ASI-SR)
Clinical Global Impression (CGI)

Intelligence (Matrigma)
Personality (NEO-FFI-3)
Health care consumption (TIC-P)
Treatment credibility/expectancy (The Credibility/Expectancy Questionnaire)
Working alliance (WAI-S)

Biological samples
Blood samples: complete blood count (Hb, MCV, TPK, LPK), liver function tests (ASAT, ALAT, GGT), alcohol marker (PEth), DNA-extraction
Cortisol in hair
Urine screening for alcohol and illicit drugs (U-Etg etc.)

Randomisation

Treatment (week 1-12, to be completed over a maximum of 20 weeks)
Assessments
PTSD-symptom severity (PCL-C)
Anxiety symptom severity (STAI)
Depression symptom severity (BDI-II)
Alcohol consumption (TLFB)
Alcohol craving (PACS)

Biological samples
Urine screening for alcohol and illicit drugs (U-Etg etc.)

Treatment
COPE or RP (Project MATCH 12 sessions)

To be complemented by:

Post sessions 1, 6 and 12
Treatment credibility/expectancy (The Credibility/Expectancy Questionnaire)
Working alliance (WAI-S)

Sessions 6 and 12
Blood samples: complete blood count (Hb, MCV, TPK, LPK), liver function tests (ASAT, ALAT, GGT), alcohol marker (PEth)

Sessions 6 (+ 0-7 days) and 12 (+ 0-7 days)
CAPS-5
CGI

Post session 12
Alcohol dependence severity (ADS, AUDIT)
Level of function in important areas of life (ASI-SR)
Intelligence (Matrigma)
Personality (NEO-FFI-3)
Health care consumption (TIC-P)
Treatment credibility/expectancy (The Credibility/Expectancy Questionnaire)
Working alliance (WAI-S)

Biological samples
Cortisol in hair

Follow up six and nine months post baseline (approximately weeks 26 and 38)
Interview and assessments
PTSD diagnosis and symptom severity (CAPS-5, PCL-C)
Anxiety symptom severity (STAI)
Depression symptom severity (BDI-II)
Alcohol consumption during the past 90 days (TLFB)
Alcohol craving (PACS)
Alcohol dependence severity (ADS, AUDIT)
Level of function in important areas of life (ASI-SR)
Clinical Global Impression (CGI)
Intelligence (Matrigma)
Personality (NEO-FFI-3)
Health care consumption (TIC-P)
Treatment credibility/expectancy (The Credibility/Expectancy Questionnaire)
Working alliance (WAI-S)

Biological samples
Blood samples: complete blood count (Hb, MCV, TPK, LPK), liver function tests (ASAT, ALAT, GGT), alcohol marker (PEth)
Cortisol in hair
Urine screening for alcohol and illicit drugs (U-Etg etc.)

Summary of study procedures and assessments

Recruitment and screening

Subjects will be recruited among treatment seeking women at the outpatient units at Capio Maria in Stockholm, the Stockholm Centre for Dependency Disorders and Linköping University Hospital, all in Sweden. Information about the study may be posted within the Stockholm County health care system, and on the web pages of Capio Maria, The Stockholm Centre for Dependency Disorders and Linköping University Hospital. Women seeking treatment for problems related to alcohol use or PTSD will be assessed for eligibility. Once informed consent is obtained baseline measures will be gathered during a series of screening visits, as detailed in Appendix 1.

Assessment in clinical practice

Trauma assessment will be carried out in routine clinical practice and the Life Events Checklist (LEC) will be used to identify any traumatic situations experienced. The M.I.N.I. International Neuropsychiatric Interview for DSM-5 (MINI) (16) will be used to establish presence of current PTSD and moderate to severe AUD, as well as absence of exclusionary diagnoses. The screening will be performed by a psychiatrist or psychologist.

Informed consent process

The consent form is attached. On the screening visit, subjects will receive additional verbal information and have an opportunity to ask questions before accepting or declining to participate in the study.

Study start

Potential study subjects will receive verbal and written information about the study by the psychiatrist. All medication must be stable for eight weeks before study start. If deemed medically appropriate and agreeable to the patient, use of medications for AUD, e.g. disulfiram, naltrexone, acamprosate and nalmefene, will be discontinued two weeks prior to baseline assessment of PTSD symptom severity and three weeks prior to COPE.

Assessment at baseline (week 0)

All subjects will be interviewed about demographics, such as marital status, highest completed education etc., as well as about their AUD and PTSD. The Alcohol Dependence Scale (ADS; (17)) and the Alcohol Use Disorder Identification Test (AUDIT; (18)) will be used to assess the severity of AUD. These are widely used self-report scales with well-established psychometric properties. Time-Line Follow-Back (TLFB; (19)) will be used to obtain quantitative drinking

measures. The TLFB use self-report to obtain estimates of daily drinking, and has been evaluated with both clinical and nonclinical populations. Using a calendar, subjects provide retrospective estimates of their daily drinking over a specified time period. Several memory aids are used to enhance recall (e.g., calendar; key dates serve as anchors for reporting drinking; standard drink conversion). The TLFB has been shown to possess good psychometric characteristics with a variety of drinker groups, and allows for generation of outcome variables that provide quantity and frequency measures of alcohol consumption. It has been used in the majority of trials evaluating pharmacotherapies for alcohol dependence, now moderate to severe AUD. The Penn Alcohol Craving Scale (PACS; (20)) is a five item self-report scale to assess alcohol cravings the preceding week. It has been used in numerous medication trials, and is sensitive to approved anticraving medications such as naltrexone. The Beck Depression Inventory - Second edition (BDI-II; (21)) and State-Trait Anxiety Inventory (22) are widely established rating scales for depression and anxiety respectively. To obtain a time line of PTSD symptom severity over the course of the study, the PTSD Check List – Civilian Version (PCL-C; (23, 24)) will be used. The PCL is a standardized self-report rating scale for PTSD comprising 17 items that correspond to the key symptoms of PTSD. PCL-C is the version of the scale that is not specific for combat-related trauma, and can be used to assess PTSD symptom severity after a range of any type of traumatic event. The self-report version of the Addiction Severity Index (ASI-SR) will be used to assess problem severity in seven different domains of functioning (medical, psychiatric, alcohol use, drug use, housing, occupation and legal). The self-report version of the ASI has been shown to produce composite scores that are highly correlated with those obtained through the use of the more extensively validated interviewer based ASI instrument (25, 26). Clinical Global Impression will be rated (CGI (27)). Assessment will also include measures of personality (NEO-FFI-3) (28), IQ (Matrigma (29)), working alliance with one’s therapist (WAI-S) (30), treatment expectations and credibility (CEQ) (31) and health care consumption (TiC-P) (32).

The Clinician-administered PTSD Scale (CAPS) will be used as the primary assessment to determine PTSD symptom severity. CAPS is a structured interview that corresponds to the DSM-5 criteria for PTSD (33, 34). It can be used to diagnose current or lifetime PTSD. In addition to assessing PTSD symptoms, questions target the impact of symptoms on social and occupational functioning, improvement in symptoms since a previous CAPS administration, overall response validity, overall PTSD severity. The CAPS was designed to be administered by clinicians and clinical researchers who have a working knowledge of PTSD, but can also be administered by appropriately trained paraprofessionals.

Laboratory tests

Blood samples will be obtained for analysis of genetic variation (e.g. CNR1, FAAH). Urine samples will be obtained for analysis of alcohol biomarkers (EtG), and to screen for illicit drug use. Blood samples will be obtained for analysis of alcohol biomarkers (GGT, AST, ALT, MCV and PEth, as well as a complete blood count). Hair samples will be obtained for analysis of cortisol.

Randomization

Karolinska Trial Alliance (KTA) will be responsible for the randomization, which will be insulated from the researchers. Subjects will be randomized in blocks, 1:1 to receive relapse prevention or COPE. When subjects are included the PI or coordinator at The Stockholm Centre for Dependency Disorders is to be contacted and to provide both the subject with the appropriate randomization number and information on whether the subject is to receive relapse prevention or COPE, information which is kept in a sealed envelope for each randomization number until inclusion, when the applicable envelope is opened and the site is informed of what treatment to give the subject.

Treatment

COPE (12, 13, 35) will be delivered by trained therapists, and consists of twelve individual 90-minute sessions. Its key elements include repeated imaginal exposure (i.e. revisiting and recounting traumatic memories) and processing of traumatic memory (i.e. discussing thoughts and feelings related to revisiting the memory and cognitive restructuring). Participant homework includes repeated listening to a recording of the recounting made during the last session, and repeated *in vivo* exposure to safe situations previously avoided because of trauma related distress. If participants demonstrate no or minimal distress when recounting the traumatic memory and confronting situations previously avoided because of trauma related anxiety, any remaining sessions will focus on other psychosocial problems.

All COPE sessions will be digitally recorded, transferred to a computer, encrypted and stored on a remote file sharing service. They will be viewed by supervisors, e.g. licensed psychologists and psychotherapists, supervising the therapists and providing feedback on their delivery of COPE. Sessions may also be viewed in supervision.

The relapse prevention, a Swedish version of the Project MATCH Cognitive Behavioral Coping Skills Therapy Manual (36), will be delivered by trained therapists. It consists of twelve 45-60-minute sessions, and includes skills such as becoming aware of and dealing with craving. COPE

and RP sessions will be digitally recorded and 10-15 percent of sessions will be monitored for fidelity.

Follow up - at each session, after session 6 and 12 and six and nine months post baseline

Prior to each COPE or RP session, each subject will fill out self-assessment forms on PTSD-symptom severity (PCL-C), alcohol consumption (TLFB), alcohol craving (PACS), anxiety symptom severity (STAI) and depression symptom severity (BDI-II). For information on follow ups at sessions 6 and 12 and six and nine months post baseline, see summary above on pages 8-10.

Blind assessors

The CAPS and CGI will be completed after sessions 6 and 12 and six and nine months post baseline. At follow-up, assessors blinded to the patient's treatment condition (COPE or RP) will conduct CAPS by telephone no later than 7 days after the last session or after the follow-up screening. This offers an additional layer of protection from accidental unblinding and enhances blinding because assessors are not physically located where patients are receiving treatment.

Management of data and specimen

Each subject will receive a unique randomization number. All data will be captured and stored in individual clinical research files (CRFs) linked to the individual through the randomization number. For the duration of the study, CRFs will be stored in a cabinet in an access-controlled area of the respective outpatient unit. Upon completion of the study, data will be transferred to an electronic database, in a format stripped of all personally identifiable information other than the randomization number. The code key linking randomization numbers to personally identifiable information will be stored in a locked cabinet with access only for the trial leader or their designee.

Therapist training and treatment adherence

All psychologists and therapists giving COPE or relapse prevention as part of the study will be trained to do so. COPE training consists of a three day course and relapse prevention training of a one day course. All staff must have a working knowledge of SUD or other psychiatric disorders and experience of treating them.

Psychiatrists, physicians and therapists are regular staff at the units trained in the methodology and will be supported in matters related to the study, the treatments etc., by the PI and research coordinator. Those giving COPE will be supervised by an experienced therapist, currently the only one certified in prolonged exposure (PE) in Sweden. Those giving RP will receive use the supervision, which is part of their routine clinical practice, for RP supervision. To evaluate treatment fidelity, i.e. to what extent the psychologist/therapist's delivery of COPE and RP corresponds to the respective manual, the COPE and RP sessions will be recorded with a digital camera, the resulting files encrypted and stored on a remote file sharing service. They will be viewed by supervisors, e.g. licensed psychologists and psychotherapists, supervising the therapists and providing feedback on their delivery of COPE. The supervisor who supervises psychologists/therapists and the person/persons responsible for the fidelity rating will have access to the material in order to ensure that the patients receive psychological treatment of the highest quality.

Subject management following completion of study

Following completion, if the study assessment identifies a remaining unmet treatment need, subjects will be encouraged to seek such treatment within or without the current clinic. Referrals to other treatment providers will be offered, should they be deemed adequate.

Stopping rules and interim analyses

The trial will be terminated if serious adverse events are encountered that prompt a re-evaluation of the risks involved, or if accrual indicates that it will not complete within a reasonable timeframe. No interim analyses are planned.

Compensation

Subjects will receive a gift certificate of 50 SEK (approximately 7 USD) after sessions 1-11 of COPE and at the completion of session 12 and the CAPS immediately thereafter. Subjects will then receive gift certificates of 150 SEK (approximately 20 USD) at follow ups six and nine months post baseline.

Information to participating staff

Participating staff will be required to read the protocol and key background literature, and document having done so. They will discuss these materials at a joint site-initiation meeting with the Principal Investigator and co-investigators, and their participation will be documented.

Staff will have quarterly meetings, and will have ongoing interactions with the Principal Investigator and co-investigator over the course of the study.

Delegation policy

All sponsor responsibilities with the exception of subject withdrawal decisions, adverse event reporting and study termination decisions will be delegated to a lead investigator at each of the participating sites.

Publication policy

Results will be submitted for publication in a peer-reviewed journal.

References

1. Benedek DM. Posttraumatic stress disorder from Vietnam to today: the evolution of understanding during Eugene Brody's tenure at the journal of nervous and mental disease. J Nerv Ment Dis. 2011;199(8):544-52.
2. Copeland WE, Magnusson A, Goransson M, Heilig MA. Genetic moderators and psychiatric mediators of the link between sexual abuse and alcohol dependence. Drug and alcohol dependence. 2011;115(3):183-9.
3. Kessler RC, Chiu WT, Demler O, Merikangas KR, Walters EE. Prevalence, severity, and comorbidity of 12-month DSM-IV disorders in the National Comorbidity Survey Replication. Arch Gen Psychiatry. 2005;62(6):617-27.
4. McCarthy E, Petrakis I. Epidemiology and management of alcohol dependence in individuals with post-traumatic stress disorder. CNS Drugs. 2010;24(12):997-1007.
5. Mills KL, Teesson M, Ross J, Peters L. Trauma, PTSD, and substance use disorders: findings from the Australian National Survey of Mental Health and Well-Being. Am J Psychiatry. 2006;163(4):652-8.
6. Yehuda R, LeDoux J. Response variation following trauma: a translational neuroscience approach to understanding PTSD. Neuron. 2007;56(1):19-32.
7. Heilig M, Egli M, Crabbe JC, Becker HC. Acute withdrawal, protracted abstinence and negative affect in alcoholism: are they linked? Addict Biol. 2010;15(2):169-84.
8. Shin LM, Rauch SL, Pitman RK. Amygdala, medial prefrontal cortex, and hippocampal function in PTSD. Ann N Y Acad Sci. 2006;1071:67-79.
9. Quirk GJ, Mueller D. Neural mechanisms of extinction learning and retrieval. Neuropsychopharmacology. 2008;33(1):56-72.
10. Sherin JE, Nemeroff CB. Post-traumatic stress disorder: the neurobiological impact of psychological trauma. Dialogues in clinical neuroscience. 2011;13(3):263-78.
11. Foa EB, Yusko DA, McLean CP, Suvak MK, Bux DA, Oslin D, et al. Concurrent Naltrexone and Prolonged Exposure Therapy for Patients With Comorbid Alcohol Dependence and PTSD A Randomized Clinical Trial. Jama-J Am Med Assoc. 2013;310(5):488-95.
12. Killeen TK, Back SE, Brady KT. The Use of Exposure-Based Treatment Among Individuals With PTSD and Co-occurring Substance Use Disorders: Clinical Considerations. J Dual Diagn. 2011;7(4):194-206.
13. Mills KL, Teesson M, Back SE, Brady KT, Baker AL, Hopwood S, et al. Integrated exposure-based therapy for co-occurring posttraumatic stress

disorder and substance dependence: a randomized controlled trial. JAMA. 2012;308(7):690-9.
14. Nationella riktlinjer för vård och stöd vid missbruk och beroende – Stöd för styrning och ledning. Edita Bobergs AB, Falun2015. p. 166.
15. Stewart SH. Alcohol abuse in individuals exposed to trauma: a critical review. Psychol Bull. 1996;120(1):83-112.
16. Sheehan DV, Lecrubier Y, Sheehan KH, Amorim P, Janavs J, Weiller E, et al. The Mini-International Neuropsychiatric Interview (M.I.N.I.): the development and validation of a structured diagnostic psychiatric interview for DSM-IV and ICD-10. J Clin Psychiatry. 1998;59 Suppl 20:22-33;quiz 4-57.
17. Skinner HA, Allen BA. Alcohol dependence syndrome: measurement and validation. J Abnorm Psychol. 1982;91(3):199-209.
18. Saunders JB, Aasland OG, Babor TF, de la Fuente JR, Grant M. Development of the Alcohol Use Disorders Identification Test (AUDIT): WHO Collaborative Project on Early Detection of Persons with Harmful Alcohol Consumption--II. Addiction. 1993;88(6):791-804.
19. Sobell MB, Sobell LC, Klajner F, Pavan D, Basian E. The reliability of a timeline method for assessing normal drinker college students' recent drinking history: utility for alcohol research. Addict Behav. 1986;11(2):149-61.
20. Flannery BA, Volpicelli JR, Pettinati HM. Psychometric properties of the Penn Alcohol Craving Scale. Alcohol Clin Exp Res. 1999;23(8):1289-95.
21. Beck AT, Steer RA, Brown GK. Manual for the Beck depression inventory-II. San Antonio, TX: Psychological Corporation; 1996. 82 p.
22. Spielberger CD, Gorsuch RL, Lushene RE. The State-Trait Anxiety Inventory: Testmanual. Palo Alto, CA: Consulting Psychologist Press; 1970.
23. Blanchard EB, Jones-Alexander J, Buckley TC, Forneris CA. Psychometric properties of the PTSD Checklist (PCL). Behav Res Ther. 1996;34(8):669-73.
24. Wilkins KC, Lang AJ, Norman SB. Synthesis of the psychometric properties of the PTSD checklist (PCL) military, civilian, and specific versions. Depress Anxiety. 2011;28(7):596-606.
25. McLellan AT, Cacciola JC, Alterman AI, Rikoon SH, Carise D. The addiction severity index at 25: Origins, contributions and transitions. Am J Addict. 2006;15(2):113-24.
26. Rosen CS, Henson BR, Finney JW, Moos RH. Consistency of self-administered and interview-based Addiction Severity Index composite scores. Addiction. 2000;95(3):419-25.
27. Guy W. Clinical global impression scale. The ECDEU Assessment Manual for Psychopharmacology-Revised Volume DHEW Publ No ADM 76. 1976;338:218-22.

28. McCrae RR, Costa PT. NEO inventories for the NEO Personality Inventory-3 (NEO-PI-3), NEO Five-Factor Inventory-3 (NEO-FFI-3), NEO Personality Inventory-Revised (NEO PI-R): Professional manual. Lutz, FL: PAR; 2010.
29. Mabon H, Sjöberg A. Matrigma. Technical manual. 2011 ed. Stockholm: Assessio International AB; 2011.
30. Tracey TJ, Kokotovic AM. Factor Structure of the Working Alliance Inventory. Psychol Assess. 1989;1(3):207-10.
31. Devilly GJ, Borkovec TD. Psychometric properties of the credibility/expectancy questionnaire. J Behav Ther Exp Psychiatry. 2000;31:73-86.
32. Bouwmans C, De Jong K, Timman R, Zijlstra-Vlasveld M, Van der Feltz-Cornelis C, Swan Tan S, et al. Feasibility, reliability and validity of a questionnaire on healthcare consumption and productivity loss in patients with a psychiatric disorder (TiC-P). BMC Health Serv Res. 2013;13(217).
33. Weathers FW, Keane TM, Davidson JR. Clinician-administered PTSD scale: a review of the first ten years of research. Depress Anxiety. 2001;13(3):132-56.
34. Blake DD, Weathers FW, Nagy LM, Kaloupek DG, Gusman FD, Charney DS, et al. The development of a Clinician-Administered PTSD Scale. J Trauma Stress. 1995;8(1):75-90.
35. Back SE, Dansky BS, Carroll KM, Foa EB, Brady KT. Exposure therapy in the treatment of PTSD among cocaine-dependent individuals: description of procedures. J Subst Abuse Treat. 2001;21(1):35-45.
36. Väckarklockan. En manual för återfallsprevention vid beroende. Solna: Meda AB; 2001.

COPE vs RP RCT statistical analysis plan 241022

One pre-selected model per outcome will be run. There will be no testing for differences at baseline.

Co-primary outcomes

Clinician-rated PTSD symptom severity

Difference in PTSD symptom severity, measured using CAPS-5, between groups up to 9-month follow-up. To be analyzed using a linear mixed model (LMM) with fixed effects for treatment, time and their interaction, a random effect for subjects and a random intercept. Restricted maximum likelihood estimation, Satterthwaite approximation, and a first-order autoregressive covariance matrix are to be used.

Data from baseline, mid-treatment (session 6) and the last treatment session (session 12), 6- and 9-month follow-ups when CAPS-5 was conducted.

Intention-to-treat (ITT) analysis will include all randomized participants, results will be presented. A modified ITT analysis will be run without the two participants who did not have current PTSD at baseline, results will be presented.

Alcohol use

Difference in grams of alcohol consumed per week, measured using Timeline Follow Back (TLFB), between groups up to 9-month follow-up. To be analyzed using an LMM with fixed effects for treatment, time and their interaction, a random effect for subjects and a random intercept. Restricted maximum likelihood estimation, Satterthwaite approximation, and a first-order autoregressive covariance matrix are to be used.

Data from baseline, sessions 1-12, 6- and 9-month follow-ups when TLFB was completed.

ITT and modified ITT as above.

Secondary outcomes

Self-rated PTSD symptom severity

Measured using the PTSD Checklist – Civilian version (PCL-C), range 17-85. To be analyzed using an LMM with fixed effects for treatment, time and their interaction, a random effect for subjects and a random intercept. Restricted maximum likelihood estimation, Satterthwaite approximation, and a first-order autoregressive covariance matrix are to be used.

Data from baseline, sessions 1-12, 6- and 9-month follow-ups when PCL-C was completed.

ITT and modified ITT as above.

A biomarker of alcohol use

A biomarker of alcohol use (PEth) in blood. To be analyzed using an LMM with fixed effects for treatment, time and their interaction, a random effect for subjects and a random intercept. Restricted maximum likelihood estimation, Satterthwaite approximation, and a first-order autoregressive covariance matrix are to be used.

Data from baseline, mid-treatment (session 6) and the last treatment session (session 12), 6- and 9-month follow-ups when blood samples were taken.

ITT and modified ITT as above.

PTSD remission

Clinician-rated PTSD remission in percent per treatment arm. Data from baseline, mid-treatment (session 6) and the last treatment session (session 12), 6- and 9-month follow-ups when CAPS-5 was conducted.

ITT.
