# Supplementary material for: Concurrent Treatment of Posttraumatic Stress Disorder and Alcohol Use Disorder in Women: A Randomized Clinical Trial
Source: JAMA Netw Open. 2025 Jul 15;8(7):e2521087. doi: 10.1001/jamanetworkopen.2025.21087 (PMC12371515; doi:10.1001/jamanetworkopen.2025.21087)
Supplement: Supplement 2. — eTable 1. Complete List of Assessments eTable 2. Clinician-Rated PTSD Remission eFigure. CONSORT Flow Diagram eReferences [file jamanetwopen-e2521087-s002.pdf]

Supplemental Online Content

Persson A, Axén Å, Capusan AJ, Magnusson Å, Heilig M. Concurrent treatment of posttraumatic stress disorder and alcohol use disorder in women: a randomized clinical trial. *JAMA Netw Open*. 2025;8(7):e2521087.
doi:10.1001/jamanetworkopen.2025.21087

eTable 1. Complete List of Assessments

eTable 2. Clinician-Rated PTSD Remission

eFigure. CONSORT Flow Diagram

eReferences

This supplemental material has been provided by the authors to give readers additional information about their work.

© 2025 Persson A et al. *JAMA Network Open*

eTable 1. Complete list of assessments

	STUDY PERIOD										
	**Enrolment**	**Allocation**	**Post-allocation, treatment sessions**							**Follow-ups, post treatment start**	
**TIME POINT**	$-t_1$	0	1	2	…	6	7	…	12	6 months	9 months
**ENROLMENT:**											
Eligibility screen[a]	X										
Informed consent	X										
Information on medication use before and during trial	X										
**Allocation:**		X									
**Interventions:**											
Integrated treatment			♦	─	─	─	─	─	♦		
Relapse prevention			♦	─	─	─	─	─	♦		
**Assessments:**											
Interview (demographic data, PTSD and alcohol use)[b]		X									
Interview (PTSD) and clinical global impression[c]		X				X			X	X	X
Self-reports (PTSD, anxiety, depression, alcohol use, craving)[d]		X	X	X	X	X	X	X	X	X	X
Self-reports (alcohol dependence severity, level of function, healthcare consumption)[e]		X							X	X	X
Self-reports (Treatment credibility/expectancy, working alliance)[f]		X	X			X			X	X	X
Psychological tests (general mental ability, personality)[g]		X							X	X	X
Blood samples (complete blood count, liver function, alcohol biomarker)[h]		X				X			X	X	X
Blood sample (DNA)		X									
Hair samples (cortisol)		X							X	X	X

© 2025 Persson A et al. *JAMA Network Open*

Alcohol and drug screening[i]		X	X	X	X	X	X	X	X	X	X

[a]The eligibility screen included the Life Events Checklist for DSM-5 (LEC-5)[1] to screen for potentially traumatic events and the Mini International Neuropsychiatric Interview for DSM-5 (MINI)[2] to assess psychiatric diagnoses, administered in routine clinical practice.
[b]An interview covering demographic data, PTSD and alcohol use was developed specifically for this trial.
[c]PTSD symptom severity and diagnosis was measured using the Clinician-Administered PTSD Scale for DSM-5 (CAPS-5)[3] conducted by trained interviewers that were blinded to treatment allocation. Clinical global impression was assessed using the Clinical Global Impression (CGI) – Severity.[4]
[d]PTSD symptom severity was also measured using the PTSD Checklist – Civilian Version (PCL-C).[5,6] Anxiety symptom severity, state anxiety, was measured using the State-Trait Anxiety Inventory (STAI).[7] Depression symptom severity was measured using the Beck Depression Inventory II (BDI-II).[8,9] Alcohol use per week was measured using Timeline Follow-back (TLFB).[10] Alcohol craving was measured using Penn Alcohol Cravings Scale (PACS).[11]
[e]AUD severity was measured using the Alcohol Dependence Scale (ADS)[12,13] and the Alcohol Use Disorder Identification Test (AUDIT).[14] The Addiction Severity Index (ASI-SR) was used to assess problem severity in seven different domains of functioning (medical, psychiatric, alcohol use, drug use, housing, occupation and legal).[15,16] The self-report version of the ASI has been shown to produce composite scores that are highly correlated with those obtained from the more extensively validated interviewer-based ASI.[15,16] Health care consumption was measured using the Treatment Inventory of Costs in Patients with psychiatric disorders (TiC-P).[17]
[f]Treatment expectations and credibility were assessed using the Credibility/Expectancy Questionnaire (CEQ).[18] Working alliance was assessed using the Working Alliance Inventory – Short (WAI-S).[19]
[g]Psychological tests were used to assess general mental ability (GMA) and personality. Matrigma was used to asses GMA.[20] Participants whose results were extremely low were offered more extensive testing using the Wechsler Adult Intelligence Scale (4th ed. (WAIS-IV))[21] to assess IQ and the exclusion criteria of IQ below 70. Personality was assessed using the NEO Five-Factor Inventory-3.[22]
All of the instruments above have good psychometric properties[1-5,7-28] and most are widely used.
[h]Alcohol use was measured using the biomarker phosphatidylethanol (PEth) in micromoles per liter.[29] PEth has shown high sensitivity and specificity for identifying alcohol use.[29] Blood samples were taken by laboratory staff blinded to treatment allocation.
[i]Substance use was measured in breath, saliva or urine samples taken by trained staff.

© 2025 Persson A et al. *JAMA Network Open*

eTable 2. Clinician-rated PTSD remission

	No. (%)					
**Treatment**	**Screening**	**Baseline**	**Session 6**	**Session 12**	**6-month follow-up**	**9-month follow-up**
**Integrated treatment**	45 (100.0)	43 (95.6)	16 (35.6)	18 (40.0)	19 (42.2)	25 (55.6)
**Relapse prevention**	45 (100.0)	45 (100.0)	9 (20.0)	14 (31.1)	15 (33.3)	17 (37.8)

Abbreviations: PTSD, posttraumatic stress disorder.

© 2025 Persson A et al. *JAMA Network Open*

eFigure 1. CONSORT flow diagram[a]

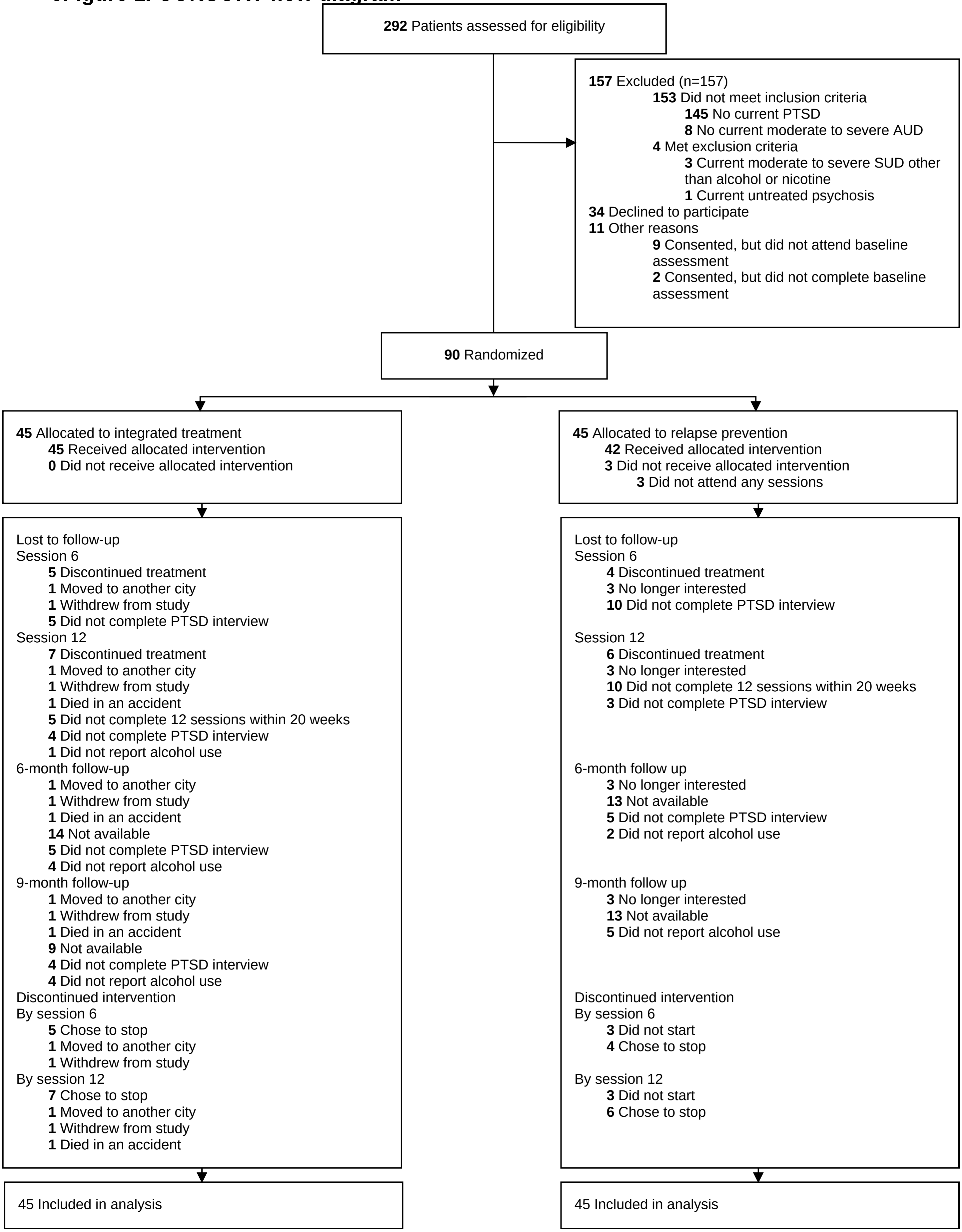

© 2025 Persson A et al. *JAMA Network Open*

a. For COPE: Providers (n=3), Number of participants per provider (median=8 [IQR=14.5, min=4, max=33]). For RP: Providers (n=3), Number of participants per provider (median=8 [IQR=15.5, min=3, max=34]).

© 2025 Persson A et al. *JAMA Network Open*

eReferences

1. Weathers FW, Blake DD, Schnurr PP, Kaloupek DG, Marx BP, Keane TM. The Life Events Checklist for DSM-5 (LEC-5). Instrument available from the National Center for PTSD at www.ptsd.va.gov. 2013.
2. Sheehan DV, Lecrubier Y, Sheehan KH, et al. The Mini-International Neuropsychiatric Interview (M.I.N.I.): The Development and Validation of a Structured Diagnostic Psychiatric Interview for DSM-IV and ICD-10. *J Clin Psychiatry.* 1998;59(Supplement 20):22-33.
3. Weathers FW, Blake DD, Schnurr PP, Kaloupek DG, Marx BP, Keane TM. The Clinician-Administered PTSD Scale for DSM-5 (CAPS-5). Interview available from the National Center for PTSD at www.ptsd.va.gov. 2013.
4. Guy W, National Institute of Mental Health (U.S.) Psychopharmacology Research Branch, Early Clinical Drug Evaluation Program. *ECDEU Assessment Manual for Psychopharmacology.* Rev. ed. Rockville, Maryland: U.S. Department of Health, Education, and Welfare, Public Health Service, Alcohol, Drug Abuse, and Mental Health Administration, National Institute of Mental Health, Psychopharmacology Research Branch, Division of Extramural Research Programs; 1976.
5. Blanchard EB, Jones-Alexander J, Buckley TC, Forneris CA. Psychometric Properties of the PTSD Checklist (PCL). *Behav Res Ther.* 1996;34(8):669-673.
6. Arnberg FK, Michel PO, Johannesson KB. Properties of Swedish posttraumatic stress measures after a disaster. *J Anxiety Disord.* 2014;28(4):402-409.
7. Spielberger CD, Gorsuch RL, Lushene RE. *The State-Trait Anxiety Inventory: Testmanual.* Palo Alto, CA: Consulting Psychologist Press; 1970.
8. Beck AT, Steer, R.A., Brown, G.K. *Manual for the Beck Depression Inventory-II.* San Antonio, TX.: Psychological Corporation.; 1996.
9. Wang YP, Gorenstein C. Psychometric properties of the Beck Depression Inventory-II: a comprehensive review. *Rev Bras Psiquiatr.* 2013;35(4):416-431.
10. Sobell LC, Sobell MB. Timeline Follow-Back: A Technique for Assessing Self-Reported Ethanol Consumption. In: Allen J, Litten RZ, eds. *Measuring Alcohol Consumption: Psychosocial and Biological Methods.* Totowa, NJ: Humana Press; 1992:41-72.
11. Flannery BA, Volpicelli JR, Pettinati HM. Psychometric Properties of the Penn Alcohol Craving Scale. *Alcoholism: Clinical and Experimental Research.* 1999;23(8):1289-1295.
12. Skinner HA, Allen BA. Alcohol Dependence Syndrome: Measurement and Validation. *J Abnorm Psychol.* 1982;91(3):199-209.
13. Skinner HA, Horn JL. *Alcohol Dependence Scale: Users Guide.* Toronto, Canada: Addiction Research Foundation; 1984.
14. Saunders JB, Aasland OG, Babor TF, De la Fuente JR, Grant M. Development of the Alcohol Use Disorders Identification Test (AUDIT): WHO Collaborative Project on Early Detection of Persons with Harmful Alcohol Consumption-II. *Addiction.* 1993;88:791-804.
15. McLellan AT, Cacciola JC, Alterman AI, Rikoon SH, Carise D. The Addiction Severity Index at 25: origins, contributions and transitions. *Am J Addict.* 2006;15(2):113-124.
16. Rosen CS, Henson BR, Finney JW, Moos RH. Consistency of self-administered and interview-based Addiction Severity Index composite scores. *Addiction.* 2002;95(3):419-425.

17. Bouwmans C, De Jong K, Timman R, et al. Feasibility, reliability and validity of a questionnaire on healthcare consumption and productivity loss in patients with a psychiatric disorder (TiC-P). *BMC Health Serv Res.* 2013;13(217).
18. Devilly GJ, Borkovec TD. Psychometric properties of the credibility/expectancy questionnaire. *J Behav Ther Exp Psychiatry.* 2000;31:73-86.
19. Tracey TJ, Kokotovic AM. Factor Structure of the Working Alliance Inventory. *Psychol Assess.* 1989;1(3):207-210.
20. Mabon H, Sjöberg A. *Matrigma. Technical manual.* 2011 ed. Stockholm: Assessio International AB; 2011.
21. Wechsler D. *Wechsler Adult Intelligence Scale.* 4th edition ed. San Antonio, TX: Pearson Assessment; 2008.
22. McCrae RR, Costa PT. *NEO inventories for the NEO Personality Inventory-3 (NEO-PI-3), NEO Five-Factor Inventory-3 (NEO-FFI-3), NEO Personality Inventory-Revised (NEO PI-R): Professional manual.* Lutz, FL: PAR; 2010.
23. Weathers FW, Bovin MJ, Lee DJ, et al. The Clinician-Administered PTSD Scale for DSM-5 (CAPS-5): Development and initial psychometric evaluation in military veterans. *Psychol Assess.* 2018;30(3):383-395.
24. Harrington T, Newman E. The psychometric utility of two self-report measures of PTSD among women substance users. *Addict Behav.* 2007;32(12):2788-2798.
25. McDowell I. *Measuring Health: a Guide to Rating Scales and Questionnaires.* New York: Oxford University Press; 2006.
26. Gray MJ, Litz BT, Hsu JL, Lombardo TW. Psychometric properties of the life events checklist. *Assessment.* 2004;11(4):330-341.
27. Horvath AO, Greenberg LS. Development and Validation of the Working Alliance Inventory. *J Couns Psychol.* 1989;36(2):223-233.
28. Hanson WE, Curry KT, Bandalos DL. Reliability Generalization of Working Alliance Inventory Scale Scores. *Educ Psychol Meas.* 2002;62(4):659-673.
29. Ulwelling W, Smith K. The PEth Blood Test in the Security Environment: What it is; Why it is Important; and Interpretative Guidelines. *J Forensic Sci.* 2018;63(6):1634-1640.

© 2025 Persson A et al. *JAMA Network Open*
