# Supplementary material for: Concurrent Treatment of Posttraumatic Stress Disorder and Alcohol Use Disorder in Women: A Randomized Clinical Trial
Source: JAMA Netw Open. 2025 Jul 15;8(7):e2521087. doi: 10.1001/jamanetworkopen.2025.21087 (PMC12371515; doi:10.1001/jamanetworkopen.2025.21087)
Supplement: Supplement 3. — Data Sharing Statement [file jamanetwopen-e2521087-s003.pdf]

Data Sharing Statement

Persson. Concurrent Treatment of Posttraumatic Stress Disorder and Alcohol Use Disorder in Women. *JAMA Netw Open*. Published July 15, 2025.
doi:10.1001/jamanetworkopen.2025.21087

Data
Additional Information: ISRCTN registry, https://www.isrctn.com, study record ISRCTN61391164
Data available: No

Additional Information
Explanation for why data not available: Individual level data cannot be shared as we have neither participant nor ethical review board consent to share it.
